# Split-Ubiquitin Based Membrane Yeast Two-Hybrid (MYTH) System: A Powerful Tool For Identifying Protein-Protein Interactions

**DOI:** 10.3791/1698

**Published:** 2010-02-01

**Authors:** Jamie Snider, Saranya Kittanakom, Jasna Curak, Igor Stagljar

**Affiliations:** Department of Biochemistry, University of Toronto; Department of Molecular Genetics, University of Toronto; Terrence Donnelly Centre for Cellular and Biomolecular Research (CCBR), University of Toronto

## Abstract

The fundamental biological and clinical importance of integral membrane proteins prompted the development of a yeast-based system for the high-throughput identification of protein-protein interactions (PPI) for full-length transmembrane proteins. To this end, our lab developed the split-ubiquitin based Membrane Yeast Two-Hybrid (MYTH) system. This technology allows for the sensitive detection of transient and stable protein interactions using *Saccharomyces cerevisiae* as a host organism. MYTH takes advantage of the observation that ubiquitin can be separated into two stable moieties: the C-terminal half of yeast ubiquitin (C_ub_) and the N-terminal half of the ubiquitin moiety (N_ub_). In MYTH, this principle is adapted for use as a 'sensor' of protein-protein interactions. Briefly, the integral membrane bait protein is fused to C_ub_ which is linked to an artificial transcription factor. Prey proteins, either in individual or library format, are fused to the N_ub_ moiety. Protein interaction between the bait and prey leads to reconstitution of the ubiquitin moieties, forming a full-length 'pseudo-ubiquitin' molecule. This molecule is in turn recognized by cytosolic deubiquitinating enzymes, resulting in cleavage of the transcription factor, and subsequent induction of reporter gene expression. The system is highly adaptable, and is particularly well-suited to high-throughput screening. It has been successfully employed to investigate interactions using integral membrane proteins from both yeast and other organisms.

**Figure:**
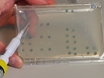


## Protocol

### 1. Background Information

Protein-protein interactions (PPIs) are the fundamental building blocks involved in governing all cellular processes. Consequently, it is essential that all interactions are tightly regulated in order to maintain cellular homeostasis, as a shift in this biological equilibrium commonly plays a role in disease and cancer cell transformation. Membrane associated proteins are amongst the most biologically important class of proteins as they can initiate complex signaling cascades, and mediate both the import and export of various molecules, including drugs, which has been of recent significance in the field of health care as problems related to drug resistance have become increasingly common. Gaining insight into the complexity of this protein class requires the identification of their interacting partners. Discovering such partners has proven challenging, as often it requires harsh conditions that must be optimized for each membrane-bound protein [1].

The fundamental biological and clinical importance of integral membrane proteins prompted the development of a yeast-based system for the high-throughput identification of PPI for full-length transmembrane proteins. To this end, we developed the split-ubiquitin based Membrane Yeast Two-Hybrid (MYTH) system [2-4]. This tool allows for the sensitive detection of transient and stable protein interactions. It has been successfully applied to study exogenous and endogenous proteins expressed in the model organism *Saccharomyces cerevisiae *[3-7]. MYTH takes advantage of the observation that ubiquitin may be separated into two moieties: the C-terminal half (C_ub_) and the N-terminal half (NubI). *in vivo* studies have shown that these moieties spontaneously reconstitute due to their high affinity for one-another (**Figure. 1a**). However, introducing an isoleucine 13 to glycine point mutation in the N-terminal half of ubiquitin (producing a fragment referred to as N_ub_G) prevents this spontaneous re-association [8] **(Figure. 1b).**

We use this principle in the MYTH system **(Figure. 1c and 2)**. Briefly, the integral membrane bait protein is fused to a C_ub_ moiety which is linked to an artificial transcription factor consisting of the *Escherichia coli* DNA-binding protein LexA and the activation domain of VP16 from herpes simplex virus. Preys are generated by fusion of cDNA or genomic DNA derived fragments to the NubG moiety. An interaction between bait and prey proteins in a yeast host leads to reconstitution of a full-length 'pseudo-ubiquitin' molecule, with subsequent recognition by cytosolic deubiquitinating enzymes (DUBs) and proteolytic release of the transcription factor. The transcription factor can then enter the nucleus of the cell, and activate a reporter gene system (typically involving expression of the *HIS3*, *lacZ* and *ADE2* genes) allowing the growth of yeast strains on selective media, which is indicative of bait and prey interaction [2-4].

The identification and characterization of integral membrane protein interactions will provide information to help fully understand their function. As we more precisely understand and dissect the roles of the proteins that interact with integral membrane proteins, we may gain insight into the dynamic interplay involved in regulating these proteins and discover novel targets that may have therapeutic potential.

### 2. Selection of Bait and Appropriate MYTH System

Prior to conducting MYTH analysis, verify that your protein has its N- and/or C-terminus in the cytosol of the cell. It is essential that the C_ub_-LexA-VP16 tag be fused to your protein at such a terminus, since the deubiquitinating enzymes necessary for transcription factor release are located in the cytosol [4].
Next, decide which of the two major variants of MYTH is suitable. For non-native yeast proteins, traditional MYTH (tMYTH) may be used, wherein baits are overexpressed ectopically from a plasmid. For native yeast proteins, integrated MYTH (iMYTH) is the method of choice. In iMYTH baits are endogenously tagged with the C_ub_-LexA-VP16 tag, leaving them under the control of their native promoter. This is advantageous, as the wild-type expression level of baits helps eliminate problems associated with protein overexpression, such as an increased number of false positives [3]. With the exception of the initial bait construction and the selective media used, both forms of MYTH are carried out in an essentially identical manner. For clarity, we will focus mainly on the use of tMYTH in this report, as this variant can, in principle, be used with membrane proteins from virtually any organism and is thus more widely applicable.

### 3. Bait Generation and Validation

Required Media and Solutions
  **Sterile ddH_2_O** prepared by autoclaving at 121°C, 15 psi for 30 minutes.
  **3-amino-1,2,4-triazole (3-AT)** solution prepared as a 1M stock solution in ddH_2_O. Sterilize by passage through a 0.2 μm filter.
  **YPAD Growth Media** consisting of 1% w/v yeast extract, 2% w/v peptone, 2% w/v glucose and 100 μM adenine prepared in ddH_2_O. Sterilize by autoclaving at 121°C, 15 psi for 30 minutes.
  **10x Amino Acid/Nucleotide Base Mix:** The complete mix contains 1.0 mM Adenine, 1.8 mM Uracil, 1.0 mM Arginine, 1.0 mM Histidine, 2.3 mM Isoleucine, 7.6 mM Leucine, 1.6 mM Lysine, 10.1 mM Methionine, 3.0 mM Phenylalanine, 16.8 mM Threonine, 2.0 mM Tryptophan, 1.7 mM Tyrosine and 12.8 mM Valine, prepared in ddH_2_O .  For drop-out omit the necessary amino acid(s) and/or nucleotide base(s). Sterilize by autoclaving at 121°C, 15 psi for 30 minutes.
  **Synthetic Drop-Out (SD) Growth Media **consisting of 0.67% w/v yeast nitrogen base (without amino acids but with ammonium sulfate), 2% w/v glucose, 2% w/v agar and 1x Amino Acid/Nucleotide Mix, prepared in ddH_2_O. Prepare both liquid and solid (containing 2% agar) SD-Leucine media. Also prepare solid SD-Tryptophan-Leucine and SD-Tryptophan-Leucine-Adenine-Histidine media. Sterilize by autoclaving at 121°C, 15 psi for 30 minutes. Pour solid media into 100x15 mm Petri dishes.
  **Synthetic Drop-Out (SD) Growth Media Containing  3-AT. **Prepare SD-Tryptophan-Leucine-Adenine-Histidine media as  described, but containing 3-AT at concentrations of 25, 50, 75 and 100 mM. Add the appropriate amount of 3-AT from the 1M sterile stock solution to the media after it has been autoclaved and cooled (but has not yet solidified). Pour into 100x15 mm Petri dishes.
  **PEG/Lithium Acetate Mix **consisting of 40% w/v PEG-3350, 120 mM Lithium Acetate and 167 μg/mL Salmon Sperm DNA (Type III Sodium Salt) prepared in ddH_2_O. To ensure the sterility of this mixture prepare it from sterile water and solutions (i.e. autoclaved 50% PEG-3350, autoclaved 1M Lithium Acetate and 2 mg/mL Salmon Sperm DNA type III sodium salt prepared in sterile ddH_2_O).
  **Enzyme and reagents for conducting PCR**.
  **Commercial miniprep kit.**
  **Soda lime glass beads (0.5 mm).**
  **Competent *Escherichia coli* cells suitable for plasmid propagation **(e.g. DH5α, XL10 gold) and standard media suitable for bacterial propagation and plasmid selection.
  **Specific yeast strains, plasmids and primers **as described in the protocol.
Generation of tMYTH Baits by Gap Repair
  The bait must be cloned into an appropriate vector for tagging and expression. A variety of tMYTH vectors are currently available for use in bait construction. Vectors such as the pCMBV4, pAMBV4 and pTMBV4 allow the construction of C-terminally tagged baits (BAIT-C_ub_-LexA-VP16) under the control the CYC1 (weak), ADH1 (strong) and TEF1 (very strong) promoters, respectively. N-terminally tagged baits (LexA-VP16-C_ub_-BAIT) can be generated using vectors such as pTLB-1 and pBT3-N, under the control of the TEF1 and CYC1 promoters, respectively. The vector choice depends upon the bait and must be determined empirically. In certain cases higher bait expression is necessary in order to detect interactions, while in other cases bait overexpression may actually be detrimental, leading to an increased number of false positives.
  Restriction digest the selected plasmid at the appropriate restriction site(s). Cleavage should occur only in the immediate vicinity of the C_ub_-LexA-VP16 tag (upstream of the tag for C-terminal tagging or downstream for N-terminal tagging).  For instance, when using the pAMBV4 vector, SfiI is an ideal choice. Store the digested plasmid at -20°C until ready for use.
  Design primers for the amplification and cloning of the gene of interest. The 5' end of your Forward Primer must match approximately 35-40 nucleotides upstream of the restriction site, while the 3' end must match the first 18-20 nucleotides of the target gene. The 5' end of the Reverse Primer must match the reverse complement of approximately 35-40 nucleotides downstream of the restriction site, with the 3' end matching the reverse complement of the last 18-20 nucleotides of the target gene (omitting the stop codon if the C_ub_-LexA-VP16 tag is being placed at the C-terminus). Depending upon whether N- or C-terminal tagging is being performed, select the 35-40 nucleotides of the Forward or Reverse primer such that the target gene is cloned in frame with the C_ub_-LexA-VP16 tag.
  Amplify the gene of interest by PCR using the above primers. PCR parameters will depend upon the particular enzyme and specific primers used.
  Transform the PCR product along with the digested plasmid into an appropriate yeast lab strain bearing a *leu2* mutation (e.g. BY4741). A MYTH reporter strain (e.g. THY.AP4 or L40) can be used but is not necessary, as the purpose of the yeast at this point is simply to serve as an environment in which the gap repair homologous recombination can occur. Transformation should be carried out as follows:
    Inoculate a single colony of your selected yeast strain into 5 mL of sterile YPAD media and incubate overnight at 30°C with constant shaking (200 rpm).
    Dilute the overnight culture into 50 mL of fresh YPAD media to an OD600 of ~0.15 and incubate at 30°C with shaking (200 rpm). Grow for approximately 3-4 hours until an OD600 of ~ 0.6 is reached.
    Centrifuge the cells at 700xg for 5 minutes and remove the supernatant.
    Resuspend the cell pellet in 25 mL sterile ddH_2_O and centrifuge at 700xg for 5 minutes.
    Remove the supernatant and resuspend the cell pellet in 1 mL sterile ddH_2_O.
    Add 100 μL of the cells, 300 μL PEG/Lithium Acetate mix, and the digested plasmid (50 fmol) and PCR product (250-500 fmol) to a microfuge tube.
    Incubate at 30°C for 30 minutes.
    Heat shock at 42°C for 1 hour.
    Centrifuge at 3000xg for 5 min and remove the supernatant.
    Resuspend the cell pellet in 200 μL sterile ddH_2_O and plate the entire volume onto solid, SD-Leucine selective media. Grow at 30°C for 2-4 days.
    Grow up a single colony of the transformed strain in 5 mL SD-Leucine liquid media at 30°C overnight.
    Centrifuge cells at 700xg for 5 minutes and remove the supernatant.
    Isolate bait plasmid DNA from the cell pellets using any commercial miniprep kit. Follow the standard protocol with one modification. In order to ensure sufficient yeast cell lysis, add a small volume of 0.5 mm soda lime glass beads to the pellet after initial resuspension and vortex vigorously for 5 minutes. Then proceed with the commercial protocol as normal.
    Transform isolated yeast DNA into a competent *E. coli* strain suitable for plasmid propagation (e.g. DH5α, XL10 Gold) with a transformation efficiency of at least 1x10^7^ cells/μg DNA. Note that bait plasmids can be selected for using kanamycin.
    Harvest plasmid DNA from the transformed *E. coli* using a standard DNA isolation method or commercial kit.
    Verify proper construction of the bait plasmid by sequencing.
    Transform the verified bait construct into an appropriate MYTH reporter strain (e.g. THY.AP4, L40). The yeast transformation protocol just described can be used, substituting the bait plasmid DNA in place of the digested plasmid and PCR product.
Bait Validation - Proper Localization
  Prior to use, bait strains are analyzed to ensure that they are properly localized to the yeast membrane. When using iMYTH, this localization will depend specifically upon the properties of the tagged bait. For tMYTH, bait plasmids generally include a signal sequence (e.g. Matα) directing the expressed protein to the plasma membrane. Localization is determined using fluorescence microscopy. Inclusion of a YFP molecule in the bait tag sequence (i.e. C_ub_-YFP-LexA-VP16) is the simplest and most direct approach, allowing direct visualization of live cells, and is commonly used in iMYTH. Alternatively, a standard immunofluorescence approach using antibody against the LexA or VP16 components of the tag can be used.
Bait Validation - N_ub_G/I Control Test
  Once proper localization of the bait has been established, it is necessary to ensure that the bait does not activate the reporter system alone or in the presence of non-interacting preys (i.e. verify that the bait is not  self-activating). This is accomplished using the N_ub_G/I Test, where the bait is transformed with interacting ( positive) and non-interacting ( negative) control preys, and growth is assessed on selective media. A bait must grow on selective media in the presence of the positive control, and not grow in the presence of the negative control, in order to be suitable for use in MYTH.
  Begin by transforming the bait strain to be tested with 100-200 ng of control prey plasmid. The previously described yeast transformation protocol can be used, substituting SD-Leucine media in place of YPAD and using SD-Tryptophan-Leucine media for the final plating step. The following control prey constructs are commonly used:
    pOST1-N_ub_I (Positive Control)
    pOST1-N_ub_G (Negative Control)
    pFUR4-N_ub_I (Positive Control)
    pFUR4-N_ub_G (Negative Control)
  OST1 is a component of the oligosaccharyl complex and is localized to the endoplasmic reticulum membrane [9] while FUR4 is a uracil permease and is localized to the plasma membrane [10].  Although these proteins are generally our first choice for use as  non-interacting preys, their suitability will vary on a case by case basis. In the unlikely event that your bait is predicted to genuinely interact with both of these controls, alternative preys will need to be selected. Recall that N_ub_I is the wild-type form of the N-terminus of ubiquitin, and spontaneously interacts with C_ub_ independent of an interaction between the proteins to which the C_ub_ and N_ub_ are fused. Thus N_ub_I preys constitute positive controls, while the N_ub_G preys (carrying the Isoleucine 13 to Glycine mutation which prevents spontaneous association of N_ub_ and C_ub_) serve as negative controls.
  Resuspend single colonies of each transformed bait into 100 μL sterile ddH_2_O.
  Serially dilute the resuspended cells in sterile ddH_2_O to produce dilutions of 1/10, 1/100 and 1/1000.
  Spot 5 μL volumes of undiluted and diluted cells onto SD-Tryptophan-Leucine and SD-Tryptophan-Leucine-Adenine-Histidine media with and without 3-AT at a range of concentrations. 3-AT acts as a competitive inhibitor of the *HIS3* reporter gene, and serves to increase the stringency of the selection process. It can be useful in some cases for inhibiting the non-specific growth of weakly to moderately self-activating baits.
  Allow the spots to dry and then incubate the plates at 30°C for 2-4 days.
  All transformants should grow on the SD-Tryptophan-Leucine plates, indicating that they have been successfully transformed with prey plasmid. Baits which do not self-activate will grow on SD-Tryptophan-Leucine-Adenine-Histidine media only when transformed with N_ub_I prey constructs, and not with N_ub_G preys. Note what concentration of 3-AT is required in the media (if any) as this will need to be used during screening.

### 4. Screening

Required Media and Solutions
  **Sterile ddH_2_O** prepared by autoclaving at 121°C, 15 psi for 30 minutes.
  **0.9% NaCl Solution** prepared in ddH_2_O and sterilized by autoclaving at 121°C, 15 psi for 30 minutes.
  **Sodium Phosphate Solution** consisting of 493 mM sodium phosphate dibasic and 250 mM sodium phosphate monobasic in ddH_2_O. Sterilize by autoclaving at 121°C, 15 psi for 30 minutes.
  **X-Gal (5-bromo-4-chloro-3-indolyl-β-D-galactopyranoside) Solution **prepared as a 100 mg/mL stock solution in N,N-dimethyl formamide.
  **2xYPAD Growth Media **containing 2% w/v yeast extract, 4% w/v peptone, 4% w/v glucose and 100 μM adenine, prepared in ddH_2_O. Sterilize by autoclaving at 121°C, 15 psi for 30 minutes.
  **Synthetic Dropout (SD) Growth Media, **prepared as described previously. Prepare liquid SD-Leucine and solid SD-Tryptophan-Leucine. Pour the solid media into both 100x15 mm Petri dishes. Prepare solid SD-Tryptophan-Leucine-Adenine-Histidine media in 150 mm round plates, 16 plates for each screen, containing 3-AT, if necessary, at the concentration determined from the N_ub_G/I control test.
  **Synthetic Dropout (SD) Media + 5-Bromo-4-Chloro-3-Indoyl-β-D-Galactopyranoside (X-Gal).** Prepare SD-Tryptophan-Leucine-Adenine-Histidine media containing agar as described previously. After autoclaving, allow to cool, add 3-AT (if necessary) followed by 1/10th volume of sterile Sodium Phosphate Solution. Next, add X-Gal Solution to a final concentration of 80 μg/mL. Mix thoroughly and pour into 150 mm round plates.
  **PEG/Lithium Acetate Solution II **containing 40% PEG-3350, 100 mM Lithium Acetate, 1 mM EDTA and 10 mM Tris pH 7.5. Prepare this solution using sterile ddH_2_O and solutions (e.g. autoclaved 50% PEG-3350, 1 M Lithium Acetate, 100 mM Tris pH 7.5 and 500 mM EDTA pH 8.0).
  **Lithium Acetate / Tris EDTA Solution **containing 110 mM Lithium Acetate, 11 mM Tris pH 7.5 and 1.1 mM EDTA. Prepare this solution using sterile ddH_2_O and solutions (e.g. autoclaved 1M Lithium Acetate, 100 mM Tris pH 7.5 and 500 mM EDTA pH 8.0).
  **10x Tris EDTA Solution** consisting of 100 mM Tris pH 7.5 and 10 mM EDTA prepared in ddH_2_O. Sterilize by autoclaving at 121°C, 15 psi for 30 minutes.
  **Single-stranded Carrier DNA (ssDNA) Solution** containing 2 mg/mL Salmon Sperm DNA Type III sodium salt, prepared in sterile ddH_2_O.
  **Commercial miniprep kit.**
  **Soda lime glass beads (0.5 mm).**
  **Competent *Escherichia coli* cells suitable for plasmid propagation **(e.g. DH5α, XL10 gold) and standard media suitable for bacterial propagation and plasmid selection.
  **Specific yeast strains and plasmids **as described in the protocol
Large Scale Transformation
  Inoculate a single colony of the MYTH reporter strain containing your bait into 5 mL of SD-Leucine media and incubate overnight at 30°C with shaking (200 rpm).
  Dilute the overnight culture into 200 mL SD-Leucine media to an OD600 = 0.15 and incubate at 30°C with shaking (200 rpm). Grow until the OD600 = 0.6 -  0.7 (approximately 4-5 hours).
  Shortly before the target OD600 is reached, thaw an aliquot of ssDNA solution. Boil at 100°C for 5 minutes and then cool on ice. Repeat once.
  When the target OD600 has been reached, harvest the cells via centrifugation at 700xg for 5 minutes (divide the 200 mL culture between 4x50 mL screw-cap centrifuge tubes).
  Wash each pellet with 30 mL sterile ddH_2_O and briefly vortex the sample. Centrifuge at 700xg for 5 minutes.
  Discard the supernatant and resuspend each pellet in 1 mL Lithium Acetate / Tris EDTA solution. Transfer to a sterile 1.5 mL microfuge tube and centrifuge at 700xg for 5 minutes.
  Discard the supernatant and resuspend each pellet in 600 μL of Lithium Acetate / Tris EDTA solution.
  Add the following to 4x15 mL screw-cap centrifuge tubes:
    2.5 mL PEG/Lithium Acetate Solution II
    600 μL resuspended cells
    100 μL ssDNA solution
    7 μg of prey library DNA
  Libraries containing preys tagged at either the N- or C-terminus with N_ub_G, and prepared from a variety of cDNA or genomic sources, are commercially available (www.dualsystems.com). The specific library used must be determined on a case-by-case basis, dependent upon the selected bait and experimental objectives.
  Vortex the tubes for 1 minute to ensure thorough mixing and then incubate in a 30°C waterbath for 45 minutes. Mix briefly every 15 minutes.
  Add 160 μL dimethyl sulfoxide (DMSO) to each tube and mix immediately by inverting the tubes.
  Incubate in a 42°C waterbath for 20 minutes.
  Collect transformants by centrifugation at 700xg for 5 minutes.
  Discard the supernatant. Recover transformants by resuspension of each pellet in 3 mL 2xYPAD. Pool all samples together in a single 50 mL screw-cap centrifuge tube.
  Incubate at 30°C for 90 minutes for cell recovery.
  Centrifuge at 700xg for 5 minutes and discard the supernatant.
  Resuspend the cell pellets in 4.9 mL sterile 0.9% NaCl.
  Using 100 μL of resuspended cells prepare 10-fold serial dilutions in sterile 0.9% NaCl ranging from 10x to 1000x.
  Plate 100 μL of the 100x and 1000x dilutions onto SD-Tryptophan-Leucine media and incubate at 30°C for 2-3 days. These plates serve as a control and are used to calculate the efficiency of the transformation.
  Equally divide the remaining 4.8 mL of resuspended cells and plate onto large (150 mm) SD-Tryptophan-Leucine-Adenine-Histidine plates, containing the necessary amount of 3-AT as determined in the N_ub_G/I test, and incubate at 30°C for 3-4 days.
  Resuspend single colonies (each representing cells containing a potential interacting bait-prey pair) in 100 μL of 0.9% NaCl and plate 5 μL aliquots onto SD-Tryptophan-Leucine-Adenine-Histidine + X-Gal media (and including 3-AT if required). Allow to grow for 2-4 days. This step serves as a second round of selective screening, and helps in the removal of false positives obtained in the initial round. Only colonies which display robust growth and a blue color are selected for further analysis.
Prey DNA Isolation and Sequencing
  Isolate plasmid DNA from the blue yeast colonies using a miniprep protocol with the modifications described previously. Be sure to grow up the cells in SD-Tryptophan media only, to select for retention of prey, but not bait, plasmids. For screens that produce a very large number of hits, a commercially available high-throughput miniprep kit may be advantageous at this point.
  Transform isolated yeast plasmid DNA into a competent *E. coli* strain suitable for plasmid propagation (e.g. DH5α, XL10 Gold) with a transformation efficiency of at least 1x10^7^ cells/μg DNA. Note that prey plasmids can be selected for using ampicillin.
  Isolate plasmid DNA from the transformed *E. coli* using a standard DNA isolation method or commercial kit. Once again, a high-throughput miniprep kit may be useful if the sample number is large. The amplification of the DNA in *E. coli* greatly increases plasmid yield and ensures that a sufficient amount of DNA is present for both sequencing and further analysis.
  Sequence the isolated plasmids using a primer complementary to sequence within the N_ub_G.
  Compile and analyze all sequencing data to assemble your preliminary list of interactors. This may be done manually, or in an automated manner using appropriate software.
Bait Dependency Testing
  After assembly of the preliminary list of interactors it is important to re-check the interactions and eliminate promiscuous preys which interact/activate the reporter system in a manner independent of bait identity. This is accomplished using the Bait Dependency Test. In this test, all of the identified interactors are transformed back into the original bait strain, as well as a strain harboring a control artificial bait consisting of a single transmembrane domain fused to the C_ub_-LexA-VP16 tag. Transformation is performed as per the standard protocol described previously, using SD-Leucine media in place of YPAD and SD-Tryptophan-Leucine solid media for the final plating step.
  Resuspend single colonies from the above transformations in 100 μL of sterile ddH_2_O and spot 5 μL volumes onto SD-Tryptophan-Leucine-Adenine-Histidine + X-Gal media (and including 3-AT if required). Plates are then incubated for 2-4 days at 30°C.  Ideally multiple transformants should be selected for each prey, and both the original bait and artificial bait should be spotted onto the same plate.
  Yeast carrying the artificial bait and prey that cause activation of the reporter system (i.e. growth and blue color) are considered promiscuous and that specific prey is removed from the list of interactors.
  Preys that cause growth and blue coloration in yeast with the bait-of-interest, but not the artificial bait, confirms this specific interaction. If, however, yeast harboring the prey and your bait-of-interest do not grow, this prey is removed from the list of interactors.
  The remaining preys constitute the complete list of interactors identified in the MYTH screen.

### 5. Further Studies

Once MYTH screening has been completed, further analyses must be performed in order to validate and determine the biological significance of the detected interactions. The specific studies to be performed will vary on a case-by-case basis, and must be determined by the individual researcher. Some common examples of follow-up work include co-immunoprecipitation experiments and deletion studies in the native organism.  Additionally, computational analysis of the obtained data can be useful for detecting patterns, and helping to identify the potential relevance and role different interactions may play. Thus, the MYTH technology serves as a powerful  first-step' towards the identification and understanding of the critical functional interactions of membrane proteins. Coupled with detailed follow-up studies and other recently developed and emerging technologies, it promises to be a valuable tool in unlocking the mysteries of the cell.


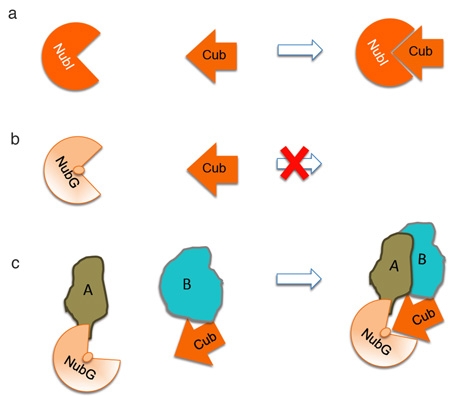
 **Figure 1. Principle of split ubiquitin.** a. Ubiquitin may be separated into two moieties: the C-terminal half (C_ub_) and the N-terminal half (N_ub_I). These moieties spontaneously reconstitute because of their high affinity for one-another. b. A N_ub_I point mutation at position 13 from an isoleucine to glycine (N_ub_G ) prevents this spontaneous re-association. c. In the MYTH system, the C_ub_ is fused to the bait-of-interest (B) and the prey is fused to the N_ub_G (A). A-B protein interaction reconstitutes pseudo-ubiquitin.


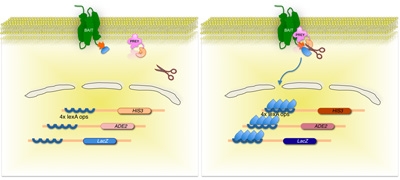
 Click here for a larger figure. **Figure 2. Split-ubiquitin based membrane yeast two-hybrid (MYTH) system.** The membrane protein-of-interest (bait) is fused to the C-terminal half of yeast ubiquitin (C_Ub_), conjugated to a transcription factor. Using a cDNA or gDNA library, each protein encoded by the library (prey) is fused to the corresponding N-terminus of the ubiquitin moiety (N_Ub_G). If the two proteins do not interact, the transcription factor remains at the membrane interface (left panel). However, if the proteins interact, the two ubiquitin moieties join, resulting in cleavage by ubiquitin specific proteases. Cleavage releases the transcription factor, resulting in expression of reporter genes (right panel)


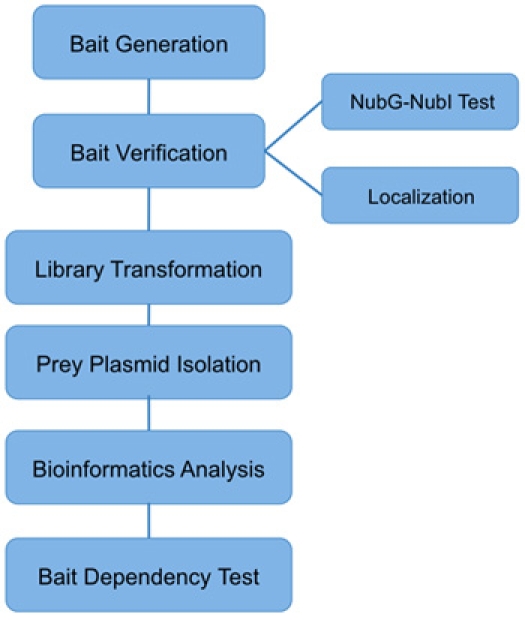
 **Figure 3. MYTH pipeline.**

## Discussion

MYTH is the first high throughput system that allows the identification of interactions between full-length membrane proteins and cytosolic or membrane-bound partners. It has been used to study membrane protein from a range of organisms [3-7]. There are, however, specific details that may need to be scrutinized to ensure the protein-of-interest is amenable to study with MYTH.

Many membrane-bound proteins are directed to the plasma membrane via a signal sequence that is subsequently cleaved to produce the mature protein. This sequence is organism-specific and it is possible that this native signal sequence will cause mis-localization of the protein-of-interest when expressed in yeast because the signal sequence remains unrecognized. To circumvent this issue, we engineered these specific proteins to be fused to the yeast signal sequence, derived from the Mating factor alpha (MATα). This peptide sequence (so-called MFα-ss) re-localizes the protein to the yeast plasma membrane and importantly, is cleaved by yeast signal peptidases. This peptide sequence is found in plasmids pTMBV-MFα and pAMBV-MFα.

Another important parameter that needs to be emphasized is bait expression levels. The promoter that drives expression of the bait regulates this parameter. It may be required to optimize bait expression levels using the NubG/NubI test and the amount of 3-AT necessary to eliminate overexpression artifacts, where the bait is "self-activating" (i.e. it promiscuously interacts with many non-specific prey proteins). Examining yeast proteins produces the most physiologically relevant bait concentrations when iMYTH is applied. In this case, the gene-of-interest is tagged with the Cub-TF within the gDNA. Alternatively, exogenous proteins can be expressed from pBT3-STE and pCMBV plasmids that carry the CYC1 promoter resulting in low bait expression. The plasmids pTMBV and pTLB1 harbor the TEF1 promoter while pAMBV has the ADH1 promoter, both that drive strong expression of the bait protein. If bait protein levels require further optimizing, it may be necessary to use the pTLB-1 plasmid that carries the TEF1 promoter, however, the LexA DNA binding domain is mutated at R156G to abate the affinity towards the exogenous reporter gene promoters, ultimately decreasing the probability of self-activation [5].

Another factor that plays an important role for MYTH success is the library of choice used for the screening process. This will depend on the endogenous bait expression profiles. For example, the bait may be expressed in specific tissues, and therefore it is important to use a library that is constructed from this specific tissue. This will ensure physiologically relevant interactions are detected.

The MYTH system is a simple and rapid tool that provides an abundance of information about a class of proteins that have been difficult to study. These identified interactions may aid in the elucidation of the full biological function of membrane proteins.
